# Phage‐resistant mutations impact bacteria susceptibility to future phage infections and antibiotic response

**DOI:** 10.1002/ece3.9712

**Published:** 2023-01-06

**Authors:** Lindsey W. McGee, Yazid Barhoush, Rafaella Shima, Miette Hennessy

**Affiliations:** ^1^ Biology Department Earlham College Richmond Indiana USA; ^2^ Department of Epidemiology and Biostatistics Drexel University Philadelphia Pennsylvania USA; ^3^ Department of Physiology and Institute of Diabetes, Obesity, and Metabolism, Perelman School of Medicine University of Pennsylvania Philadelphia Pennsylvania USA

**Keywords:** bacteriophage, *Escherichia coli*, lipopolysaccharide, pleiotropy, trade‐offs

## Abstract

Bacteriophage (phage) therapy in combination with antibiotic treatment serves as a potential strategy to overcome the continued rise in antibiotic resistance across bacterial pathogens. Understanding the impacts of evolutionary and ecological processes to the phage‐antibiotic‐resistance dynamic could advance the development of such combinatorial therapy. We tested whether the acquisition of mutations conferring phage resistance may have antagonistically pleiotropic consequences for antibiotic resistance. First, to determine the robustness of phage resistance across different phage strains, we infected resistant *Escherichia coli* cultures with phage that were not previously encountered. We found that phage‐resistant *E. coli* mutants that gained resistance to a single phage strain maintain resistance to other phages with overlapping adsorption methods. Mutations underlying the phage‐resistant phenotype affects lipopolysaccharide (LPS) structure and/or synthesis. Because LPS is implicated in both phage infection and antibiotic response, we then determined whether phage‐resistant trade‐offs exist when challenged with different classes of antibiotics. We found that only 1 out of the 4 phage‐resistant *E. coli* mutants yielded trade‐offs between phage and antibiotic resistance. Surprisingly, when challenged with novobiocin, we uncovered evidence of synergistic pleiotropy for some mutants allowing for greater antibiotic resistance, even though antibiotic resistance was never selected for. Our results highlight the importance of understanding the role of selective pressures and pleiotropic interactions in the bacterial response to phage‐antibiotic combinatorial therapy.

## Graphical abstract

We describe how exposure to bacteriophages results in the evolution of *E. coli* phage‐resistance, and this resistance is conferred to related phages that the bacteria were never exposed. Evolved phage‐resistance may come at a cost of antibiotic resistance, but the interactions are mutation and antibiotic context‐dependent.

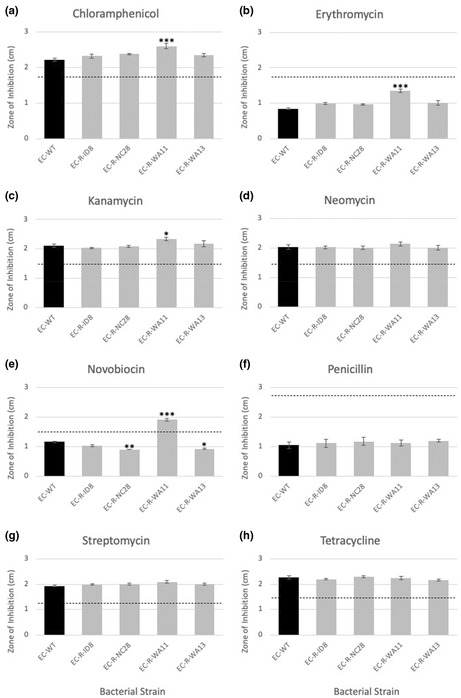

## 1 INTRODUCTION

The discovery and distribution of antibiotics has drastically reduced mortality due to infectious disease over the last 80 years. The widespread and oftentimes inappropriate use of antibiotics has led to the evolution of multidrug resistant (MDR) pathogenic bacteria. More than 2.8 million antibiotic‐resistant infections occur in the United States each year resulting in more than 35,000 deaths (Center for Disease Control and Prevention, 2021). If measures are not taken to curb the antibiotic resistance trajectory, worldwide MDR infection mortality may exceed 10 million by 2050 (O'Neill, 2014), which demands prompt development of alternatives to antibiotic usage. Bacteriophage (phage) therapy was utilized before the discovery and widespread distribution of penicillin, and with the emergence of MDR bacteria, the interest in phage therapy has sparked again (Lin et al., 2017), with the understanding that phage resistance could also arise in pathogenic bacterial populations. Studies investigating a combinatorial approach of phages and antibiotics have provided promising outcomes (Aslam et al., 2019; Tagliaferri et al., 2019); however, the interactions between evolutionary factors involved in phage and antibiotic resistance and their role in bacterial evolution remain unsettled due to their synergistic nature. Previous work has identified such interactions between phage resistance and antibiotic resistance, but the nature of the effect is dependent on the specific genotypes of bacteria and phage studied (Burmeister & Turner, 2020; German & Misra, 2001; Scanlan et al., 2015). Better understanding the impacts of evolutionary and ecological processes to the phage‐antibiotic‐resistance dynamic could advance the development of combinatorial therapy to combat MDR bacterial infections.

Specificity toward bacterial hosts offers a unique advantage of phage therapy over antibiotic therapy. A more targeted therapeutic approach could reduce negative impacts on commensal flora (Ganeshan & Hosseinidoust, 2019), and could relieve selective pressures for antibiotic resistance to arise in bacteria not targeted by the treatment. Such host specificity comes from interactions between phage proteins and host cell receptors. Bacteriophages initiate the infection of host cells through adsorption where interactions between binding proteins of the bacteriophage and bacterial cell surface receptors help recognize a sensitive host to inject genetic material (Bertozzi Silva et al., 2016). Researchers have identified several bacterial cell surface receptors involved in the adsorption process. In Gram‐negative bacteria, lipopolysaccharide (LPS), outer membrane proteins, pili, and flagella are typical receptors for phage attachment and entry. On the other hand, in Gram‐positive bacteria, common receptors include peptidoglycan, teichoic acids, and polysaccharides exposed on the bacteria's surface (Dowah & Clokie, 2018). Many bacterial strains evolve phage resistance through developing mechanisms to prevent this adsorption (Rostøl & Marraffini, 2019), and while clinicians could theoretically allow phage to co‐evolve in tandem with their bacterial hosts within a patient, the unpredictability of phage “winning” the co‐evolutionary battle is of concern. Therefore, researchers have turned toward a combinatorial strategy of antibiotics and phage to combat MDR infections.

Pleiotropy occurs when a single gene influences multiple traits on a phenotypic level (Paaby & Rockman, 2013). Synergistic pleiotropy occurs when a single gene or mutation improves two or more traits, whereas antagonistic pleiotropy occurs when beneficial effects on a focal trait are accompanied by deleterious effects on others (Cooper & Lenski, 2000; Magwire et al., 2010; Wenger et al., 2011). Trade‐offs resulting from antagonistic pleiotropic interactions have traditionally been used to explain the biological phenomena of senescence (Hughes et al., 2002; Promislow, 2004; Williams, 1957), but could also be applied to other study areas, such as niche expansion (Duffy et al., 2006; Kassen, 2002; MacLean et al., 2004; Orr, 2000; Remold, 2012), and niche construction (Chisholm et al., 2018). Evolutionary interactions between antibiotic resistance and phage resistance can be pleiotropic in effect oftentimes resulting in bacterial trade‐offs. Phage entry mechanisms use the same structures employed in other bacterial processes, therefore, mutations in these mechanisms to avoid phage infection could impact these bacterial processes, such as antibiotic response (Burmeister & Turner, 2020). By understanding the evolutionary trade‐offs between phage and antibiotic challenge to MDR bacteria, we could theorize that targeted phage therapy of MDR bacteria could be employed until phage resistance arises. Many studies have shown the occurrence of synergistic pleiotropy when microbes are exposed to differing yet simultaneous (or fluctuating) selective pressures (Burmeister & Turner, 2020; Hall et al., 2019; McGee et al., 2016; Moulton‐Brown & Friman, 2018; Sackman & Rokyta, 2019). Therefore, we theorize that phage and antibiotic therapy in sequence, not simultaneously, to avoid the occurrence of phage‐antibiotic‐resistant phenotypes. If we predict that phage‐resistance comes at a cost of antibiotic resistance, it would be beneficial to begin with phage therapy until the infection either clears or phage‐resistance arises followed by a subsequent antibiotic challenge to the more susceptible bacteria to clear the pathogen from the human host.

We selected for phage resistant mutations to arise in *Escherichia coli* strain *C* cultures by exposing wild‐type *E. coli* to four different, but closely related, lytic bacteriophage strains in the Microviridae family. Phage‐resistant mutations arose in response to infection for each phage strain in each *E. coli* culture. We then infected the phage‐resistant *E. coli* with phages it was not previously exposed to determine whether mutations conferred a correlated response to selection. We hypothesize that resistance to one phage that utilizes specific cell surface receptors for adsorption would confer resistance to other phages with overlapping adsorption methods. We then challenged phage‐resistant *E. coli* mutants with 8 different antibiotics to determine interactions between phage‐resistance and antibiotic‐resistance phenotypes to determine the prevalence of antagonistic pleiotropy. We conducted full‐genome sequencing of each *E. coli* culture to identify mutations that could be attributed to the phage‐ and antibiotic‐resistant phenotypes.

## 2 MATERIALS AND METHODS

### 2.1 Bacterial growth and conditions

We grew bacteria in lysogeny broth (LB) with 10 g tryptone, 5 g yeast extract, and 10 g/L NaCl supplemented with 2 mM CaCl_2_. LB plates included 15 g/L agar, or 7.5 g/L for top agar. For liquid cultures, overnight incubation was performed at 200 rpm shaking at 37°C. For agar cultures, overnight incubation was performed in a cabinet incubator at 37°C.

### 2.2 Bacteriophage and bacterial strains

All assays were conducted with *Escherichia coli* strain *C* and 4 bacteriophage strains, ID8, NC28, WA11, and WA13. The phages are all members of the Microviridae family, and are closely related: ID8 (G4‐like clade), NC28 and WA13 (WA13‐like clade), and WA11 (ΦX174‐like clade) (Rokyta et al., 2006). These phage strains are characterized by a circular, single‐stranded DNA genome of roughly 5 kilobases encoding 11 genes with nonenveloped, tailless capsids, icosahedral geometry. All of these phage strains grow in laboratory conditions in *Escherichia coli C* cultures at 37°C. Further genetic characterization and phenotypic assays have been conducted on these phage strains (McGee et al., 2016; Rokyta et al., 2006;2009).

### 2.3 Isolating phage‐resistant *E. coli*

Independent *Escherichia coli C* cultures were grown from isolated colonies. We infected cultures of *Escherichia coli C* with 4 bacteriophage strains, ID8, NC28, WA11, and WA13, until resistant bacterial colonies emerged to each phage. *E. coli C* cultures were grown in LB broth at 37°C to approximately 10^8^ CFU/ml. *E. coli* was mixed with ~10^6^ phage (one phage strain per culture) in top agar and grown at 37°C for 24 h. Individual bacterial colonies were picked from the plate from within the lytic zone. Each colony was confirmed as phage‐resistant *E. coli* to their respective phage via spot assay (see spot assay methods below). We archived freezer stocks of each mutant in 20% glycerol, stored at −80°C.

### 2.4 Phage infectivity spot assays

We tested whether each phage‐resistant *E. coli C* mutant was susceptible to infection by other bacteriophages to which it was not previously exposed. We acquired isolates of phage‐resistant *E. coli* mutants to each of the four phages, ID8, NC28, WA11, and WA13, and plated lawns with 100 μl of each bacterial culture. We conducted spot tests by adding ~2000 virions of each phage in 2 μl droplets to each bacterial lawn and incubating at 37°C for 24 h (Carlson, 2005). Clear zones indicating viral lysis of bacterial cells were scored as (2) complete clearing, no turbidity; (1) partial clearing, opaque or turbidity; and (0) no clearing. All assays were conducted in triplicate on each plate and across triplicate plates.

### 2.5 Antibiotic assays

**FIGURE 1:**
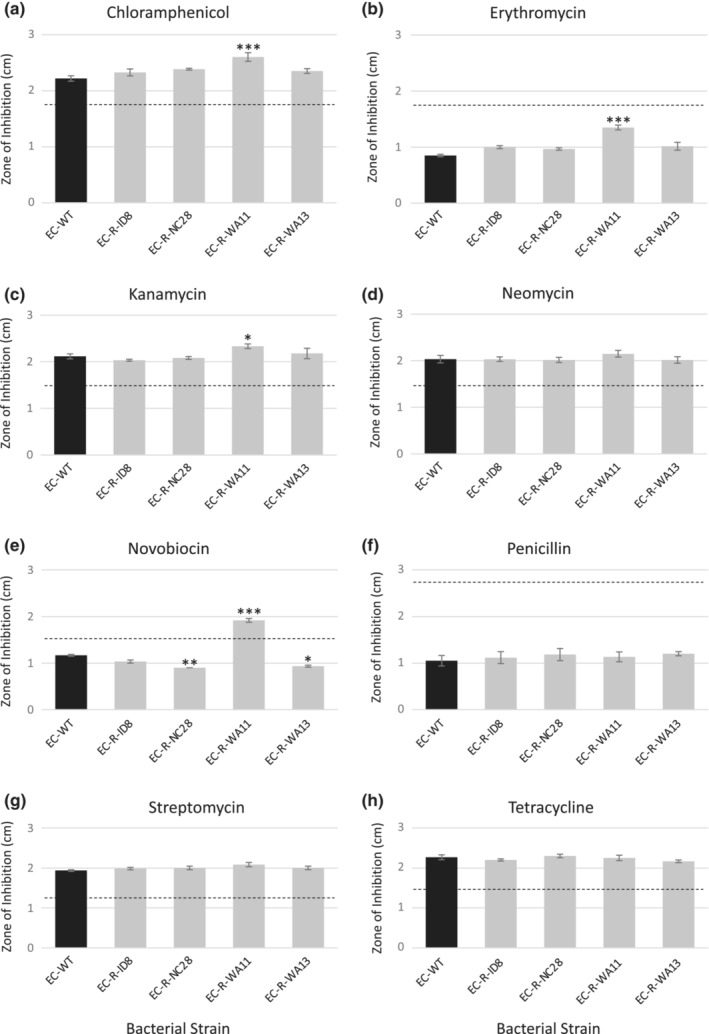
Antibiotic challenge against phage‐resistant *Escherichia coli* C mutants. Phage‐resistant mutants were challenged with antibiotic discs and compared with wild‐type *E. coli C*. the phages include ID8 (G4‐like clade), NC28 and WA13 (WA13‐like clade), and WA11 (ΦX174‐like clade). Dashed lines indicate the threshold for antibiotic resistance based on the BD BBL antibiotic susceptibility test disc kit. Antibiotics used include (a) chloramphenicol, (b) erythromycin, (c) kanamycin, (d) neomycin, (e) novobiocin, (f) penicillin, (g) streptomycin, (h) tetracycline. Assays were conducted with six replicates and statistical tests were done through pairwise contrasts with Dunnett test to correct for multiple comparisons. Error bars represent standard error of the mean.

Kirby‐Baur tests were conducted for each phage‐resistant *E. coli C* and WT *E. coli C*. Bacterial lawns were inoculated with 100 μl of each bacterial mutant. The antibiotics challenged on each bacterial mutant were chloramphenicol, erythromycin, streptomycin, kanamycin, penicillin, neomycin, novobiocin, and tetracycline. Three antibiotic discs of each antibiotic tested were placed onto two replicate bacterial mutant plates for a total of six replicates for each antibiotic assay (Carolina #805081). Plates were incubated at 37°C for 24 h. At 24 h, the diameter of the zone of inhibition was measured for each antibiotic disc. Estimates for the level of antibiotic resistance as indicated by dashed lines in Figure 1, which were determined based on the provided information from BD BBL™ Sensi‐Disc™ Antimicrobial Susceptibility Test Disc kit.

### 2.6 Genome sequence analysis

DNA was extracted from bacterial cultures using the Qiagen DNeasy Ultraclean Microbial Kit (12224–50). DNA samples were sent to the at IU Center for Genomics and Bioinformatics where the Nanopore barcoded DNA library was prepared and sequenced with a Nanopore Flow Cell. Reads were assembled with Canu (Koren et al., 2017), assembled contigs were circularized with Circulator, and VarScan was then used to call the SNPs compared with the wild‐type *E. coli* C strain (Koboldt et al., 2012).

### 2.7 Statistical analyses

Statistical analyses were conducted in R (R Core Team, 2021). A one‐way ANOVA was used to determine the overall effects of antibiotic, resistance, and the interaction on the response variable, zone of inhibition. The emmeans package allows for post hoc comparisons between groups after a fitting a model (Lenth, 2019). These subsequent pairwise contrasts were conducted to compare the zone of inhibition of *E. coli* WT (control group) to each phage‐resistant *E. coli* mutant (treatment group) for each antibiotic. This set of comparisons can be requested via trt.vs.ctrl. A Dunnett's test was used to correct for multiple comparisons, which is the default multiple comparisons adjustment used by the emmeans package.

## 3 RESULTS AND DISCUSSION

### 3.1 Phage‐Resistance carryover due to a correlated response to selection

To determine whether phage resistance toward one strain of Microviridae bacteriophage conferred resistance to other related phages, we challenged each phage‐resistant *E. coli* mutant to bacteriophages that were not previously encountered. We hypothesized that resistance to one phage that utilizes specific cell surface receptors for adsorption would confer resistance to other phages with overlapping adsorption methods. We found that bacteria exposed to one phage strain developed resistance to not only that strain but also other related strains that it had not been previously exposed (Table 1).

**TABLE 1:** Phage infectivity spot assays

	Phage ID8	Phage NC28	Phage WA11	Phage WA13
*E. coli* Wild‐type	2	2	2	2
*E. coli*‐R‐ID8	0	0	0	0
*E. coli*‐R‐NC28	0	0	0	0
*E. coli*‐R‐WA11	0	0	0	0
*E. coli*‐R‐WA13	0	0	0	0

*Note*: Spot assays to determine phage infectivity were conducted on wild‐type and phage‐resistant *E. coli* cultures. Clear zones indicating viral lysis of bacterial cells were scored as (2) complete clearing, no turbidity; (1) partial clearing, opaque or turbidity; and (0) no clearing. Virus infectivity only occurred on wild‐type *E. coli C*, indicated by lysis zones on the agar plate. For the phage‐resistance *E. coli* strains, phage infectivity was inhibited for all phage strains.

To determine the underlying genetic factors contributing to the phage‐resistant phenotype, *E. coli* mutants were sequenced using next‐generation sequencing technology and single nucleotide polymorphisms (SNPs) differing from wild‐type *E. coli* strain *C* were identified (Table 2). All 4 phage‐resistant *E. coli* strains (EC‐R‐ID8, EC‐R‐NC28, EC‐R‐WA11, and EC‐R‐WA13) contained mutations that affect lipopolysaccharide (LPS) structure and/or synthesis. LPS covers the surface of the outer membrane in Gram‐negative bacteria. Each LPS molecule contains lipid A, core oligosaccharides, and, in some bacteria, a highly variable O‐antigen component. All four *E. coli* phage‐resistant strains contained a non‐synonymous point mutation, L544F (TTG → TTT), in the *yciM* gene. *yciM* contributes to cell wall integrity by regulating LPS biosynthesis. The *yciM* gene encodes a tetratricopeptide repeat that regulates the biosynthesis of lipid A, an essential component of Gram‐negative LPS structure (Bateman et al., 2021; Mahalakshmi et al., 2014). In addition to the *yciM* mutation, strains EC‐R‐ID8 and EC‐R‐WA13 both had a nonsense point mutation, Q18* (CAG → TAG) in the *rfaH* gene. The *rfaH* protein interacts with the RNA polymerase and works to inhibit Rho‐dependent transcriptional termination (Svetlov et al., 2007). By inhibiting transcriptional termination, operons involved with LPS synthesis are expressed. Therefore, the Q18* (CAG → TAG) would impair LPS synthesis, which could result in lack of receptors for phage absorption. Lastly, in addition to the *yciM* mutation, strain EC‐R‐WA11 also had the point mutation H160L (CAC → CTC) located in the *rfaP* gene. The *rfaP* protein is involved in the pathway for LPS core biosynthesis as part of the bacterial outer membrane (Bateman et al., 2021; Pagnout et al., 2019). Mutations in the LPS structure and synthesis may have disabled all strains that use LPS as a receptor for absorption mechanisms from gaining entry to the host cell. This resistance carryover would be of concern for the implementation of phage therapy as resistance developed toward one phage strain could impact the effectiveness of an entire bacteriophage family. In order to plan effectively, a phage arsenal would need to contain phages that can target the same pathogenic host cell, but utilize different absorption mechanisms.

**TABLE 2:** Genome analysis of resistant *E. coli* mutants compared with wild‐type *E. coli C*.

Bacterial strain	Nucleotide position	Nucleotide substitution	Amino acid substitution	Gene	Gene function
EC‐R‐ID8	2,531,684	C → A	L544F (TTG → TTT)	yciM	Lipopolysaccharide synthesis
	4,485,909	C → T	Q18* (CAG → TAG)	rfaH	Lipopolysaccharide synthesis
EC‐R‐NC28	2,531,684	C → A	L544F (TTG → TTT)	yciM	Lipopolysaccharide synthesis
EC‐R‐WA11	75,889	A → T	H160L (CAC → CTC)	rfaP	Lipopolysaccharide core heptose(I) kinase
	2,531,684	C → A	L544F (TTG → TTT)	yciM	Lipopolysaccharide synthesis
EC‐R‐WA13	2,531,684	C → A	L544F (TTG → TTT)	yciM	Lipopolysaccharide synthesis
	4,485,909	C → T	Q18* (CAG → TAG)	rfaH	Lipopolysaccharide synthesis

### 3.2 Phage‐antibiotic‐resistance trade‐offs and pay‐offs

We then determined whether phage‐resistant trade‐offs exist when challenged with different classes of antibiotics. Antibiotics used include chloramphenicol, erythromycin, kanamycin, neomycin, novobiocin, penicillin, streptomycin, and tetracycline, allowing us to test across different mechanisms of action and differences in inherent susceptibility to the antibiotic (indicated by dashed lines in Figure 1). A larger zone of inhibition around the antibiotic disc indicates more susceptibility to the antibiotic. Only 1 out of the 4 phage‐resistant *E. coli* strains yielded trade‐offs between phage and antibiotic resistance. The strain where trade‐offs were detected, EC‐R‐WA11, exhibited loss in antibiotic resistance across 4 of the 8 antibiotics tested. EC‐R‐WA11, with mutations in both the yciM and rfaP genes, resulted in greater antibiotic susceptibility to chloramphenicol (*p* < .0001), erythromycin (*p* < .0001), kanamycin (*p* = .04), novobiocin (*p* < .0001) compared with wild‐type (Figure 1). Phage‐resistance due to mutations in LPS structure have been previously identified and determined to increase sensitivity to antibiotics, particularly in the rfa operons.

*RfaP* mutations often result in severely truncated LPS core regions, and this phenotype has been shown to be hypersensitive to hydrophobic antibiotics, such as chloramphenicol, kanamycin, erythromycin, and novobiocin (Chang et al., 2010; Pagnout et al., 2019). *RfaP* proteins catalyze the phosphorylation of heptose I in the bacterial outer membrane. When *rfaP* is absent or inhibited, lack of phosphorylation of heptose I results in greater permeability of the membrane allowing for entry of hydrophobic antibiotics into the cell (Yethon et al., 1998). Once inside the cell, chloramphenicol, kanamycin, and erythromycin inhibit bacterial protein synthesis. Novobiocin inhibits DNA gyrase in bacteria, but has been shown to exhibit poor bacteriocidal activity against Gram‐negative pathogens (Gellert et al., 1976). In fact, novobiocin has been proposed to be used in combination with polymyxins, such as colistin, due to their synergistic activity against bacteria (Mandler et al., 2018). Polymyxins disrupts the outer membrane of Gram‐negative bacteria, and novobiocin further impacts the bacterial outer membrane by binding to LptB, which is an ATPase that powers LPS transport to deliver LPS to the cell surface. Researchers found that novobiocin‐polymyxin together are more potent than novobiocin alone (Mandler et al., 2018). Therefore, the synergy we uncovered here may be due to mutated LPS structure as a result of phage infection followed by lack of LPS maintenance as a result of novobiocin binding to LptB.

Surprisingly, the other 3 phage‐resistant mutants (EC‐R‐ID8, EC‐R‐NC28, EC‐R‐WA13) gained antibiotic resistance to novobiocin compared with wild‐type *E. coli*, with only strains EC‐R‐NC28 and EC‐R‐WA13 being significantly more resistant (*p* = .007 and .02, respectively; Figure 1). Therefore, a mutation in *yciM* alone, or the combination of *yciM* and *rfaH* mutants, result in a pay‐off due to synergistic pleiotropy in which mutations conferring phage resistance also allows for greater antibiotic resistance, even though antibiotic resistance was never selected for. This finding was unexpected due to susceptibility of LPS mutants with defects in the basal core to hydrophobic antibiotics, such as novobiocin. However, other LPS mutants, in particular mutations not affecting the core polysaccharide, have been found to maintain the permeability barrier that can still inhibit novobiocin entry into the cell (Nobre et al., 2015). It is possible that the *yciM* and *rfaH* mutants make entry across the outer membrane even more difficult for novobiocin, resulting in greater phage and antibiotic resistance.

Limitations of this study include isolating a single phage‐resistant colony per bacteriophage infection. Future studies should evaluate additional colonies that arose due to bacteriophage infection to examine different paths to phage resistance.

## 4 CONCLUSIONS

Understanding the evolutionary implications of phage‐antibiotic‐resistance dynamics is crucial for advancing treatment against MDR bacteria. Here we show that a resistance carryover across bacteriophages that are closely related and use the same method of entry into a bacterial cell. To implement phage therapy effectively, we need to consider the development of phages that can target the same pathogenic host cell, but utilize different absorption mechanisms. Additionally, our results highlight the importance of understanding the role of selective pressures and pleiotropic interactions in the bacterial response to phage‐antibiotic combinatorial therapy. More work is needed to understand these complicated evolutionary responses, in particular to avoid the evolution of multidrug and multi‐phage resistant pathogens.

## AUTHOR CONTRIBUTIONS

**Lindsey W. McGee:** Conceptualization (lead); formal analysis (equal); funding acquisition (lead); writing – original draft (equal); writing – review and editing (equal). **Yazid Barhoush:** Investigation (equal); writing – original draft (equal); writing – review and editing (equal). **Rafaella Shima:** Formal analysis (equal); methodology (equal); writing – original draft (equal). **Miette Hennessy:** Investigation (equal); methodology (equal); writing – original draft (equal); writing – review and editing (equal).

## CONFLICT OF INTEREST

The authors declare that they have no conflict of interest.

## Data Availability

Any data not made available directly in the manuscript are available at Science Data Bank and NCBI. Bacterial genome data are available in the NCBI BioSample database (http://www.ncbi.nlm.nih.gov/biosample/) under accession numbers SAMN31581070, SAMN31581071, SAMN31581072, SAMN31581073, SAMN31581074.
